# Hypertension Management Among Hospitalised Patients at Kamenge University Hospital in Bujumbura

**DOI:** 10.24248/eahrj.v8i1.747

**Published:** 2024-03-28

**Authors:** Eugene Ndirahisha, Ramadhan Nyandwi, Joseph Nyandwi, Sebastien Manirakiza, Patrice Barasukana, Thierry Sibomana, Elysee Baransaka

**Affiliations:** a University of Burundi, University Hospital of Kamenge, Department of Cardiology; b University of Burundi, University Hospital of Kamenge, Department of Laboratory; c University of Burundi, University Hospital of Kamenge, Department of nephrology; d University of Burundi, University Hospital of Kamenge, Department of Imagery; e University of Burundi, University Hospital of Kamenge, Department of Neurology; f University of Burundi, University Hospital of Kamenge, Department of Pneumology

## Abstract

**Background:**

According to the World Health Organization in 2015, 40 million out of the 56 million deaths recorded worldwide (70%) were due to non-communicable diseases. These were mainly cardiovascular diseases, cancers, chronic respiratory diseases and diabetes.

**Methods:**

A prospective descriptive study was conducted from October 2018 to March 2019. Availability of the files in the department's archives store for medical records and availability of trained physicians to perform diagnosis and treatment of HBP were the criteria used to select departments to be included in the study.

**Results:**

Patients data were recorded from internal medicine department (59.8%), emergency department (18.1%) gynaecology and obstetrics department (13.3%) and surgery department (8.6%). The mean age of the patients who were hospitalised in the study period was 54 years (SD±10.2) with extremes of 18 and 104 years. The modal class was the age group of 50 to 60 with 24.4% of cases. Among patients who were hospitalised, 3.6% (127) had essential hypertension, of which 57.4% (73) were women.

**Conclusion:**

Notable percentage of patients hospitalized at the University Hospital of Kamenge had essential hypertension. However, patients' knowledge of their hypertensive status had no positive contribution to its management.

## BACKGROUND

Non communicable disease (NCD) such as cardiovascular disease is a set of disorders affecting the heart and blood vessels, which includes among others coronary heart disease that affect blood vessels which supply blood to the heart muscle, cerebrovascular diseases that affect vessels which supply blood to the brain, rheumatic heart disease affecting muscle and heart valves as a result of rheumatic fever, and deep vein thrombosis and pulmonary embolism.^1,2^

According to the World Health Organization (WHO) in 2015, 40 million out of the 56 million deaths recorded worldwide (70%) were due to NCDs. These were mainly cardiovascular diseases, cancers, chronic respiratory diseases and diabetes. Each year, 15 million people, aged between 30 and 69 years, die of a non-communicable disease; more than 80% of these “premature” deaths occur in low and middle income countries. Between the ages of 30 and 70, these people are at the age of maximum economic productivity. The deaths and disabilities caused by NCDs therefore have an adverse impact on development.^3^

The major risk factors unanimously recognized and common to the four main NCDs are: smoking, harmful use of alcohol, excessive consumption of salt and sugar, elevation of blood pressure and total cholesterol in the blood, insufficient physical activity, various infections (HIV, HBV, HCV, HPV), environmental pollution, obesity and an unhealthy diet. These risk factors go hand in hand with globalization, urbanization and the aging of the population which is gradually spreading to the whole of planet earth. If nothing is done to curb NCDs, a sharp increase in mortality related to these conditions will hit Africa and jeopardize all development efforts.^3,4^

Cardiovascular diseases are the leading cause of mortality in the world; with more than 17 million deaths in 2015.^5^ More than three quarters of these deaths occur in low and middle income countries including Burundi, and this poses a substantial burden in terms of mortality in hospital settings.^6^ Hypertension, or high blood pressure (HBP), is the largest contributor worldwide to CVD events but other risk factors such as tobacco use, harmful use of alcohol, diabetes and hyperlipidemia also increase its risk and related mortality.^6,7^

Burundi would be hit by cardiovascular diseases at least as much as other low income countries. Global observation of the causes of death in the community and hospitals matches well with the demographic distribution of the population. However, the country lack of data on cardiovascular diseases and their risk factors. According to a survey carried out in the Province of Kirundo in 2013, the prevalence of hypertension was 25.2% and that of its related risk factors included 0.4% (obesity), 3.1% (overweight), 20% (smoking), 88% (alcohol consumption), 91.5% (low fruit and vegetable consumption) and 16.7% (low physical activity). The situation is happening in an environment where the access to health services is a challenge.^8^ According to ministry of health in Burundi, for cardiovascular diseases, accessibility to health services is 45%.^9^

The estimations of the WHO in 2018, show that chronic NCDs in Burundi were responsible for 32% of all deaths where cardiovascular diseases (CVD) were responsible for 12% of cases followed by cancer (7%), chronic respiratory diseases (2%) and diabetes (1%). On the other hand, the risk of premature death between the age of 30 and 70 from non-communicable diseases is estimated at 23%.^3,10^ The organization of the fight against these diseases is based on prevention and care at all levels of the health system. The harmful effects caused by Chronic NCDs have their origins in the contributing factors. The burden of risk factors for NCDs in Burundi is poorly known and probably underestimated due to poor quality of data.^9^

High blood pressure being a first risk factor of cardiovascular mortality, remains a major public health problem around the world despite enormous progress made in recent years in management of HBP.^11–14^ According to the literature,^1,15,16^ 50 to 70% of symptomatic thromboembolic events occur in a hospital environment in patients discharged from medical services. It has been known since the past 30 years that antihypertensive therapy reduces cardiovascular complications, and many scientific societies have been issuing recommendations for management of hypertension.^11,17,18^ However, hypertension remains insufficiently detected, treated and controlled. In 2014, hypertension ranked 10^th^ in the ranking of causes of death in hospitals according to data from the National Directorate for Health Information System. In 2018 it was one of the main cause of morbidity in the health center of Burundi with no less than 26,564 new cases of hypertension. Permanent advances in hypertension management make it possible to push back the limits, to better prevent and live better with hypertension. However, it is known if these recommendations are applicable everywhere. Thus, in absences of a protocol and data on the prescription of antihypertensive drugs in Burundi, it seemed useful to us to carry out a study and discover the most common prescribed drugs for essential hypertension.

## METHODS

### Study Site and Design

The University Hospital of Kamenge is a tertiary referral and teaching hospital in Bujumbura, economic capital of Burundi. It is one of the 6 national hospitals in Burundi. A prospective descriptive study was conducted from October 2018 to March 2019. Availability of the files in the department's archives store for medical records and availability of trained physicians to perform diagnosis and treatment of HBP were the criteria used to select departments to be included in the study. Therefore, among the many departments of the University hospital of Kamenge, department of internal medicine, surgery, gynecology and obstetrics, and emergency were selected.

The study was to provide data on hypertension characteristics, treatment and outcome during the period of hospitalization. Apart from interviews, patients' hospital files which contained patients' past medical history, medical notes and high blood pressure measurement were reviewed.

### Inclusion criteria

Adult patients (aged 18 years and above) admitted to The University Hospital of Kamenge for HBP and receiving antihypertensive drugs.

### Sample Size and Sampling

From October 2018 to March 2019, 3506 patients had been admitted in the four departments of the University Hospital of Kamenge. Of those, 127 patients had essential hypertension, making a prevalence of 3.62%.

Demographic data, past history of hypertension and cardiovascular risk factors, clinical and para clinical findings were recorded. Interview was necessary to supplement the information already obtained from consultation registers and patient follow-up sheets. These data were transcribed on data collection sheet developed for this purpose.

### High Blood Pressure Measurement

The European hypertension association and WHO guidelines were used for evaluation of cardiovascular risk and strategies of treatment.^1,19,20^

### Data Analysis

Continuous variables were expressed as means and standard deviations and categorical variables as frequencies and percentages. These were computed using the SPSS statistical software package. Means comparisons were made using Student's t-test. Percentages were compared using Pearson's Chi Square test. A *P* value of <.05 was considered to be significant.

### Ethical Approval

The study was approved by the institutional bioethics committee of the faculty of medicine of the University of Burundi and the University Hospital of Kamenge (number Ref/FM/CE/02/11/2018). In addition, prior to conduct the study, an official authorization was issued by the University Hospital of Kamenge. Patient consented to participate after receiving information on the value of the study and that collected data will not be shared to the third party.

## RESULTS

Patients data were recorded from internal medicine department (59.8%), emergency department (18.1%) gynecology and obstetrics department (13.3%) and surgery department (8.6%). The mean age of the patients who were hospitalised in the study period was 54 years (SD±10.2) with extremes of 18 and 104 years. The modal class was the age group of 50 to 60 with 24.4% of cases.

Among patients who were hospitalized, 3.6% (127) had essential hypertension, of which 57.4% (73) were women.

Hospitalised patients who were diagnosed to have essential hypertension were adults with more than 45 years (63.7%), diabetics (43.3%) and alcohol drinkers (40.9%). Among the female patients with essential hypertension, 23.6% were pregnant (Table 1).

**TABLE 1:** Profile of Patients' Risk Factors

CVRF	All (N=127)	Men (*n*=55)	Women (*n*=72)
Age ≥ 45 years	81 (63.7)		
Alcohol	52 (40.9)		
Sedentarity	12 (9.4)		
Diabetes	49 (38.5)		
Pregnancy	NA	NA	17 (23.6)
Tobacco	26 (20.4)		
Stress	7 (5.5)		
Contraceptive	4 (3.1)		
Obesity	4 (3.1)		
Dyslipidemia	1 (0.7)		
Proteinuria	19 (14.9)		

CVRF: cardiovascular risk factors; NA = Not applicable

Among the 127 hypertensive patients, 100 (78.7%) knew that they have a history of HBP but they did not take antihypertensive drugs regularly. Management of HBP during hospitalization included hygienic and dietetic measures and taking antihypertensive drugs. Six classes of medication were used to treat hypertension as shown in the Table 2.

**TABLE 2:** Frequency of Use of Different Classes of Antihypertensive Drugs

Antihypertensive class	Number	Percentage
CAAH	75	59
Diuretics	57	44.7
ACEi	25	19.6
ARB	22	17.3
Betablocker	16	12.5

ACEi: Angiotensin converting enzyme inhibitor; ARB: Angiotensin II receptor blocker; CAAH: Centrally acting anthypertensive

All the 23 patients registered in the emergency department received injectable clonidine as antihypertensive drug. In internal medicine, hydrochlorothiazide was the most prescribed drug (31.5%) followed by losartan (28.9%) and furosemide (27.6%). The most prescribed drugs in the department of surgery were calcium channel blockers (36.3%) which included nifedipine, and angiotensin converting enzyme inhibitor (ACEi) 27.2% which was captopril. Methyldopa and clonidine were the most prescribed drugs in gynecology and obstetrics department (82.3%). In general, calcium channel blockers were frequently prescribed in the surgical department (45.4%).

Commonly, patients received monotherapy (44.8%), followed by bi-therapy (24.4%) and triple therapy (20.3%). Four drugs were used in 7% of cases and five molecules in 3.1% of the patients. Among the 57 patients who received one drug, clonidine was the most used (45.6%) followed by nifedipine (17.5%) and amlodipine (10.5%).

The main combinations were losartan hydrochlorothiazide and nifedipine clonidine with a frequency of 12.9% each. Twenty-six patients had received triple therapy and most commonly prescribed combination included nifedipinecaptopril-methyldopa (19.2%). Nine patients received four molecules and the most used combination was clonidine-amlodipine-methyldopa-furosemide (22.2%). For patients who received 5 molecules; there was not a dominant combination of drugs.

## DISCUSSION

Despite the availability of antihypertensive drugs, the control of hypertension remains insufficient.^18,19,22^ According to literature, to control hypertension, the physicians use five classes of antihypertensive drugs classes (diuretics, Calcium channel blocker, Angiotensin converting enzyme inhibitor (ACEi), beta-blockers and Angiotensin II receptor blocker (ARB) apart from modifying certain behaviors in patients.^23–26^ In this study, physicians used five classes of antihypertensive drugs including central hypertensive drugs which are usually not used in the treatment of hypertension. Indeed, clonidine is the only injectable antihypertensive drug available in Burundi.

Initial management of hypertension should take in account risk factors for cardiovascular disease and other illnesses that may amplify the impact of hypertension.^18,27,28^ In this study, the most represented cardio vascular risk factors were advanced age, male sex and alcohol use in line with the literature.^19,24,29^ In fact, George MG et al^17^ reported that advanced age is a risk factor (46%) for hypertension. Another study reported that being male (41.5%) places a person at risk of hypertension.^19^ Although 78.7% of patients knew their hypertensive history, they were considered as new patients when hospitalized because most of them did not see any importance of taking medication in absence of pain.

Hypertension control remains insufficient today despite a multitude of available antihypertensive drugs in the market and most of them at good prices.^15,19,30^ For this, fixed combinations of antihypertensive drugs should be used to improve treatment acceptability, drug adherence, blood pressure control and reduction of cardiovascular complications.^1,2,15,31,32^ In our study, the use of monotherapy corresponded to the period of treatment's initiation or trial and error, but two, three, four and five dugs were used to effectively control HBP.^23–26,33^

Types of prescribed drugs varied according to department to which the patient is admitted. All 23 patients registered in the department of emergencies received injectable clonidine which is the only injectable hypertensive substance available to treat emergencies in the University Hospital. In most cases, only hydrochlorothiazide was administered, probably because the drug provide long term effect at a lowest cost.^19,30^ In the gynecologyobstetrics department, methyldopa and clonidine were the most prescribed drugs because pregnancy limits the use of certain drugs.^15,34^

## CONCLUSION

Notable percentage of patients hospitalized at the University Hospital of Kamenge had essential hypertension. However, patients' knowledge of their hypertensive status had no positive contribution to its management. Most of the hypertensive patients were adults of more than 45 years, men, alcohol drinkers and diabetics. Types of prescribed hypertensive medication was largely influenced by the admission department. Some patients received drugs which are not recommended due to conditions they have.

### Recommendations

Raise public awareness on the importance of preventing hypertension, and controlling its risk factors. The hospital need to expand the range of antihypertensive drugs, especially injectable forms. It is recommended to elaborate the national best practice guidelines on the management of cardiovascular diseases.
